# A systematic review of compositional analysis studies examining the associations between sleep, sedentary behaviour, and physical activity with health indicators in early childhood

**DOI:** 10.1186/s44167-022-00012-2

**Published:** 2023-02-01

**Authors:** Samah Zahran, Carson Visser, Amanda Ross-White, Ian Janssen

**Affiliations:** 1 School of Kinesiology and Health Studies, Queen’s University, Kingston, ON Canada; 2 Health Sciences Library, Queen’s University, Kingston, ON Canada; 3 Department of Public Health Sciences, Queen’s University, Kingston, ON Canada

**Keywords:** Physical activity, Sedentary behaviour, Sleep, Young children, Time-use data

## Abstract

**Background:**

This systematic review examined if the composition of time spent in sleep, sedentary behaviour, and physical activity of different intensities is associated with health and developmental indicators in children aged 0–5 years.

**Methods:**

Four electronic databases (MEDLINE, EMBASE, PsycINFO, and SPORTDiscus) were searched in January 2022. Studies were eligible for inclusion if they were peer-reviewed, the average age of participants was < 6 years, and compositional data analysis was used to examine the associations between the composition of time spent in movement behaviours and health and developmental indicators.

**Results:**

Eight studies (7 cross-sectional, 1 prospective cohort) of < 2070 unique participants were included. Only a single study included children < 3 years old and 37% of the associations examined in the literature were based on indicators of body composition. The 24-h movement behaviour composition was associated with mental health indicators (3 of 4 associations examined in the literature), motor skills and development (6 of 7 associations), and physical fitness (3 of 3 associations). Reallocating time from light physical activity into moderate-to-vigorous physical activity was favourable for motor skills and development. Reallocating time from light physical activity into sleep was unfavourable for mental health. Reallocating time from light physical activity into sedentary behaviour or sleep was favourable for motor skills and development.

**Conclusions:**

This review provides some evidence that the composition of movement behaviours is important for the health of young children. Future research should consider including infants and toddlers, larger sample size, and measures of health and development other than body composition. (PROSPERO registration no.: CRD42022298370.)

**Supplementary Information:**

The online version contains supplementary material available at 10.1186/s44167-022-00012-2.

## Background

Early childhood is a critical period for physical, emotional, and social development [1], as well as adopting healthy movement behaviours including sleep, sedentary behaviour (SED), and physical activity (PA) [1–3]. Systematic reviews examining movement behaviours in isolation concluded that adequate levels of sleep, high levels of PA, and low levels of SED benefit several aspects of physical, cognitive, and social-emotional health and development in children 5 years old or younger [3–5]. Furthermore, some evidence indicates that combinations of movement behaviours influence health and developmental indicators in young children [6]. Specifically, a combination of adequate sleep, high PA, and low SED is favourably associated with motor development and physical fitness [6].

Most previous studies examining the health effects of combinations of movement behaviours used non-compositional movement behaviour exposure variables, such as the absolute time spend in sleep, PA, and SED [6]. Movement behaviours should not be treated as non-compositional variables because they are co-dependent variables that form a composition that makes a finite 24-h day [8]. Therefore, changing time in any movement behaviour must result in an equal but opposite change in time spent in one or more of the remaining movement behaviours [8]. For example, increasing sleep by 30 min per day will lead to a 30 min decrease in some combination of SED and PA.

Compositional data analysis (CoDA) statistical techniques are suitable for data that are codependent and compositional, such as the combined time spent in sleep, SED, and PA across the 24-h day [8, 9]. Recent research that used CoDA suggests that the composition of movement behaviours may influence health at all ages [10–13]. A systematic review published in 2020 that examined the association between 24-h movement behaviours and health indicators across the lifespan only included two studies that examined children of the early years [13]. CoDA is a new form of statistical analysis within the movement behaviour literature, with an exponential increase in the number of published studies in recent years [13]. Therefore, an updated and focused comprehensive review of CoDA studies examining the association between movement behaviours and health indicators in young children is warranted.

The purpose of this paper was to conduct a systematic review that considered whether the composition of time spent in sleep, SED, and PA of different intensities is associated with health and developmental indicators in children of the early years. The review also considered whether changes in the movement behaviour composition (e.g., compositional isotemporal substitutions) is associated with changes in health and developmental indicators.

## Methods

### Protocol and registration

This systematic review is registered with the International Prospective Register of Systematic Reviews (PROSPERO registration no. CRD42022298370). It was conducted per the Preferred Reporting Items for Systematic Reviews and Meta-Analyses (PRISMA) statement for reporting systematic reviews and meta-analyses [14].

### Eligibility criteria

To assist the search process and identify key study concepts a priori, the Participants, Intervention/Exposure, Comparisons, Outcomes, Study design (PICOS) framework was used [15].

### Population

The population of interest was children of the early years including infants, toddlers, and preschoolers. Studies were only included if the average age of participants was < 6 years.

### Intervention/exposure

The exposure of interest was the composition of time spent in sleep, SED, and PA of different intensities (e.g., light physical activity (LPA) and moderate-to-vigorous physical activity (MVPA)). These movement behaviours were based on the measures and definitions used by the authors of the different studies. In general, a cut-point of < 1.5 metabolic equivalents (METs) during waking hours defined SED, a range of 1.5–2.99 METs defined LPA, and a cut-point of ≥ 3.0 METs defined MVPA [16]. Only studies that used a CoDA statistical approach and had at least one measure of sleep, one measure of SED, and one measure of PA were included. No limits were placed on the methods used to assess the movement behaviours (e.g., device-based or parental-report), the follow-up length in longitudinal studies, or the intervention length in intervention studies.

### Comparator/control

The comparator was different levels and compositions of time spent in sleep, SED, and PA of different intensities. In addition, changes to the composition of movement behaviours were considered, including changes observed in an intervention setting and changes estimated from observational data (i.e., compositional isotemporal substitutions).

### Outcome(s)

All health and developmental indicators were included in this review.

### Study designs

All quantitative study designs were eligible except for reviews, meta-analyses, and case studies.

### Information sources and search strategy

A research librarian with expertise in systematic review searching created the electronic search strategy. The following databases were searched: MEDLINE, EMBASE, PsycINFO (all using the Ovid platform), and SPORTDiscus (EbscoHost). Searches were conducted on January 1, 2022, and dated back to 2015, as that is when the first study on the relationship between the 24-h movement-behaviour composition and health was published [10]. Studies were eligible for inclusion if they were published in English and peer reviewed. Grey literature (e.g., book chapters, dissertations, conference abstracts) was excluded. An example search strategy based on Ovid MEDLINE is in the Additional file 1.

### Study selection

To remove duplicate records and facilitate screening, bibliographic records were imported into Covidence software (Veritas Health Innovation, Melbourne, Australia). In level 1 screening, titles and abstracts were screened by two reviewers. Records that were not screened out by both reviewers proceeded to level 2 screening. In level 2, full-text articles were obtained and examined by two 2 reviewers. Any discrepancies about final inclusion were resolved by discussion between the two reviewers. In some cases, a third reviewer was included to resolve disputes or address uncertainties.

### Data extraction

A customized Microsoft Excel spreadsheet was used during data extraction. Data extraction was completed by one reviewer and checked for accuracy by another. Information was extracted about the study and sample characteristics, intervention/exposure, health and developmental indicator(s), results (including whether these differed by sex/gender, race/ethnicity, and socioeconomic status), and confounding variables controlled for in statistical models. Reviewers were not blinded to the authors or journals when extracting data. Results were extracted from the most fully adjusted models for studies that reported findings from multiple models. Findings were considered statistically significant at p < 0.05.

### Risk of bias assessment

A modified version of the Downs and Black checklist was used to assess the risk of bias [17]. This checklist has 27 items that assess the strength of reporting, external validity, internal validity (bias and confounding), and power. Since none of the eligible studies for our review were interventions, we removed 10 checklist items that are not relevant for observational studies (items 8, 13, 14, 16, 19, 21–24, and 26). We also modified 6 items (items 4, 5, 9, 10, 12, and 15) and added 1 item to better align with observational studies designs. The added item assessed the methods used to measure the movement behaviours (e.g., device-based or parental-report). Each item was assigned a score of 1 if the article met the quality criteria, or a score of 0 if it did not. A maximum of 18 points for prospective studies and 17 points for cross-sectional studies could be achieved. In addition, overall study quality levels were assigned based on the total number of points as follows: excellent (16–18), good (13–15), fair (10–12), or poor (≤ 9) [13, 18, 19]. For more details on these modifications and the scoring system, see the Additional file 1: Table S1. Risk of bias assessments were duplicated by 2 reviewers and any disagreements were resolved by a third.

### Synthesis of results

When CoDA is used to analyze the association between movement behaviours with health indicators, up to 17 parameters are generated for each indicator examined. These parameters include: (1) a result that reflects whether the 24-h movement behaviour composition as a whole is associated with the health indicator; (2) 4 results that reflect whether the relative time spent in each of MVPA, LPA, SED, and sleep are associated with the health indicator; and (3) 12 results that reflect whether different time reallocations are associated with changes in the health indicator (e.g., reallocating time from MVPA to LPA, reallocating time from MVPA to SED, reallocating time from MVPA to sleep, etc.).

To simplify and standardize the presentation of findings for all health and developmental indicators across all studies, we used the approach previously described in a systematic review of CoDA studies examining movement behaviours and health indicators in adults [20]. Specifically, the results of each of the up to 17 individual parameters of interest for each health and developmental indicator for each study were presented as an upward arrow (↑), a sideways arrow (↔), or a downward arrow (↓) in a summary table. For results based on the 24-h movement behaviour composition, ↑ denoted a result that was statistically significant while ↔ denoted a result that was not statistically significant. For results based on the relative contributions of each movement intensity, and results for the time substitutions, ↑ denoted the presence of a result that was statistically significant and favourable for health (e.g., relative time spent in MVPA was associated with a lower BMI), ↔ denoted the presence of a result that was not statistically significant (e.g., relative time spent in sleep was not associated with BMI), and ↓ denoted the presence of a result that was statistically significant and unfavourable for health (e.g., relative time spent in SED was associated with a higher BMI).

The ↑/ ↔ /↓ rating system was also used to summarize the overall pattern of results for each health and developmental indicator across all 8 studies. This process started by applying scores of 1, 0, and − 1 to the ↑, ↔, and ↓ ratings from the individual studies. These scores were summed and divided by the total number of ratings. For results based on the 24-h movement behaviour composition, the potential range for the final calculated value was 0 to 1. The overall pattern was rated ↑ when the final value was ≥ 0.66 or ↔ when the final value was ≤ 0.65. For the results based on the relative contributions of each movement behaviour and the time substitutions, the potential range for the final calculated value was − 1 to 1. The overall pattern was rated ↑ when the final value was 0.33 or higher, ↔ when the final value was between − 0.32 and 0.32, or ↓ when the final value was − 0.33 or lower.

Meta-analyses were planned for the time substitution findings if enough studies used comparable time reallocation estimation approaches on the same indicators. However, there were too few studies for any given health indicators and considerable heterogeneity in time reallocation approaches that deterred us from conducting meta-analysis.

## Results

### Description of studies

**Fig. 1:**
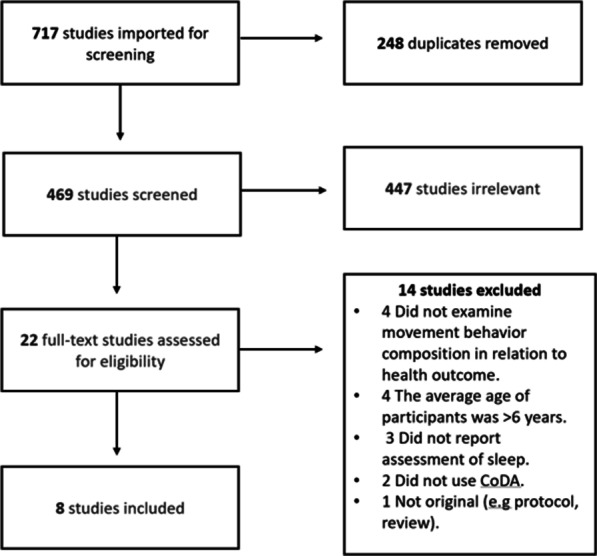
PRISMA flow diagram

The PRISMA diagram is in Fig. 1. A total of 717 studies were identified through the database searches (MEDLINE, n = 243; EMBASE, n = 229; CINAHL, n = 194; SPORTDiscus, n = 51). After duplicates were removed, there were 469 unique studies. After titles and abstracts were screened in level 1, 22 full-text articles were obtained for level 2 screening. Eight studies passed level 2 screening and were included in the systematic review [21–28]. The top two reasons for excluding studies during level 2 screening were that studies did not examine the association between the movement composition with a health and/or developmental indicator (n = 4), and an average age > 5 years (n = 4).

The characteristics of the 8 studies included in the review are in Table 1. The samples ranged from a small convenience sample of 95 participants [24] to a nationally representative sample of 552 [28]. All but one study was limited to participants aged 3 or older. Data across studies involved a total of < 2070 unique participants; there was an overlap of participants in two studies [21, 23]. All studies used an observational design; 7 were cross-sectional studies [21–25, 27, 28] and one was a prospective cohort study that included both cross-sectional and longitudinal analyses [26]. SED, LPA, and MVPA were measured using either an Actical or ActiGraph accelerometer. Six studies used a parental-report measure to estimate sleep duration [21–23, 25, 27, 28] while two used a device-based measure [24, 26].

**Table 1:** Description of studies included in the systematic review

Authors (year)	Participant characteristics			Study design	MVPA	LPA	SED	Sleep	Health and developmental indicators
	Country	Sample size	Age (y)						
Carson et al. (2017)	Canada	552	3–4	Cross-sectional	5 d Actical	5 d Actical	5 d Actical	Parent-reported	waist circumference, BMI
Taylor et al. (2018)	New Zealand	380	1–5	Cross-sectional and prospective	5–7 d Actical	5–7 d Actical	5–7 d Actical	5–7 d Actical and parent-reported	BMI, bone mineral content, bone mineral density
Bezerra et al. (2020)	Brazil	123	3–5	Cross-sectional	7 d Actigraph	7 d Actigraph	7 d Actigraph	Parent-reported	executive function
Kuzik et al. (2020)	Canada	95	3–5	Cross-sectional	7 d Actigraph	7 d Actigraph	7 d Actigraph	7 d Actigraph	BMI, motor skills, response inhibition, working memory, vocabulary, self-regulation, prosocial behaviour
Lemos et al. (2021)	Brazil	270	3–5	Cross-sectional	7 d Actigraph	7 d Actigraph	7 d Actigraph	Parent-reported	cardiorespiratory fitness, speed-agility, lower-body strength
McGee et al. (2019)	Canada	158	5.5	Cross-sectional	7 d Actical	7 d Actical	7 d Actical	Parent-reported	BMI, obesity skinfold thickness, body fat, fat-free mass
Mota et al. (2020)	Brazil	204	3–5	Cross-sectional	7 d Actigraph	7 d Actigraph	7 d Actigraph	Parent-reported	motor skills and development
St.Laurent et al. (2020)	United States	288	2.8–5.9	Cross-sectional	7 d Actigraph	Actigraph 7 d	7 d Actigraph	Parent-reported	sleep efficiency, nap frequency, sleep disturbances, bedtime resistance

*BMI* body mass index, *LPA* light physical activity, *MVPA* moderate-to-vigorous physical activity, *SED* sedentary time

Of the 8 studies, four examined the movement behaviour composition in relation to body composition measures including the body mass index, waist circumference, skinfold thickness, fat mass or % fat, and fat-free mass. One study assessed associations between the movement behaviour composition and measures of physical fitness including cardiorespiratory fitness, speed-agility, and lower-body muscular strength. One study examined measures of bone health. Two studies examined mental health (e.g., executive function, response inhibition, working memory, and vocabulary). Finally, motor development and sleep health were each assessed in a single study.

Results were not presented for all of the parameters of interest in many of the studies. Five studies did not report whether relative time spent in each movement behaviour was associated with health and developmental indicators [21–23, 25, 27], 1 study did not report findings for any time substitutions [28], and 2 studies reported the results for some time substitutions but not others [22, 24]. Of the 7 studies that showed results for time substitutions, 3 used the method explained by Dumuid et al. in 2019 [24, 26, 27, 29], 2 used the method explained by Chastin et al. in 2015 [10, 21, 22], and 2 used both of these methods [23, 25].

### Risk of bias and quality assessment

The risk of bias assessment scores for each Downs and Black checklist item and an overall quality score for each study are presented in Additional file 1: Table S1. The total checklist score of the prospective cohort study was 13.5/18 and it was rated as good quality. The total checklist scores of the 7 cross-sectional studies ranged from 11.5/17 to 12.5/17 and they were all rated as fair quality.

### Data synthesis

The associations between the 24-h movement behaviour composition with specific health and developmental indicators from the 8 individual studies are shown in Additional file 1: Table S2. Additional file 1: Table S3 and Table S4 contain the results for each of the time substitutions, which reflect the estimated changes in the health indicators that would occur if equivalent time was reallocated from one intensity of movement into another (e.g., decreasing SED by 30 min per day while increasing sleep by 30 min per day).

### Movement behaviour composition

A summary of the associations between the 24-h movement behaviour composition and health and developmental indicators is provided in Tables 2 and 3. The 24-h movement behaviour composition was significantly associated with body composition in only 1 of 20 associations examined in the literature. None of the associations for indicators of social-emotional development (0 of 7) or bone health (0 of 8) were significant. For sleep health, 2 of 4 associations were significant. Most of or all associations for the 24-h movement behaviour composition were significant for indicators of mental health (3 of 4), motor skills and development (6 of 7), and physical fitness (3 of 3).

**Table 2:** Summary of results for each health and developmental indicator

Movement behaviour component	Health and development indicator					
	Body composition	Mental health	Motor skills and development	Social-emotional development	Fitness	Sleep
Composition						
24-h composition	↑(1) ↔ (19)	↑(3) ↔ (1)	↑(6) ↔ (1)	↑(0) ↔ (7)	↑(3) ↔ (0)	↑(2) ↔ (2)
Relative time in MVPA	↑(0) ↔ (19)	↑(0) ↔ (3)	↑(3) ↔ (1)	↑(1) ↔ (6)		
Relative time in LPA	↑(2) ↔ (16) ↓(1)	↑(0) ↔ (3)	↑(0) ↔ (2) ↓(2)	↑(0) ↔ (7)		
Relative time in SED	↑(2) ↔ (17)	↑(2) ↔ (1)	↑(0) ↔ (4)	↑(0) ↔ (7)		
Relative time in sleep	↑(1) ↔ (17) ↓(1)	↑(0) ↔ (3)	↑(0) ↔ (4)	↑(0) ↔ (7)		
Time reallocation						
MVPA to LPA	↑(0) ↔ (7) ↓(0)	↑(1) ↔ (1) ↓(0)	↑(0) ↔ (3) ↓(3)	↑(0) ↔ (1) ↓(2)	↑(0) ↔ (9) ↓(0)	↑(0) ↔ (4) ↓(0)
LPA to MVPA	↑(0) ↔ (7) ↓(0)	↑(0) ↔ (2) ↓(0)	↑(3) ↔ (3) ↓(0)	↑(1) ↔ (2) ↓(0)	↑(0) ↔ (9) ↓(0)	↑(0) ↔ (4) ↓(0)
MVPA to SED	↑(0) ↔ (6) ↓(1)	↑(0) ↔ (2) ↓(0)	↑(0) ↔ (5) ↓(1)	↑(0) ↔ (2) ↓(1)		↑(1) ↔ (2) ↓(1)
SED to MVPA	↑(1) ↔ (6) ↓(0)	↑(0) ↔ (2) ↓(0)	↑(0) ↔ (6) ↓(0)	↑(1) ↔ (2) ↓(0)	↑(3) ↔ (6) ↓(0)	↑(1) ↔ (2) ↓(1)
MVPA to sleep	↑(0) ↔ (7) ↓(0)	↑(1) ↔ (1) ↓(0)	↑(0) ↔ (6) ↓(0)	↑(0) ↔ (2) ↓(1)		↑(0) ↔ (4) ↓(0)
Sleep to MVPA	↑(0) ↔ (7) ↓(0)	↑(0) ↔ (1) ↓(1)	↑(0) ↔ (6) ↓(0)	↑(1) ↔ (2) ↓(0)	↑(3) ↔ (6) ↓(0)	↑(0) ↔ (4) ↓(0)
LPA to SED	↑(0) ↔ (6) ↓(1)	↑(0) ↔ (2) ↓(0)	↑(4) ↔ (2) ↓(0)	↑(0) ↔ (3) ↓(0)		↑(0) ↔ (4) ↓(0)
SED to LPA	↑(1) ↔ (6) ↓(0)	↑(0) ↔ (1) ↓(1)	↑(0) ↔ (2) ↓(4)	↑(0) ↔ (3) ↓(0)	↑(0) ↔ (9) ↓(0)	↑(0) ↔ (4) ↓(0)
LPA to sleep	↑(1) ↔ (7) ↓(1)	↑(0) ↔ (0) ↓(1)	↑(4) ↔ (2) ↓(0)			↑(1) ↔ (1) ↓(2)
Sleep to LPA	↑(0) ↔ (7) ↓(0)	↑(0) ↔ (1) ↓(0)	↑(0) ↔ (2) ↓(4)		↑(2) ↔ (4) ↓(3)	↑(2) ↔ (1) ↓(1)
SED to sleep	↑(0) ↔ (7) ↓(0)	↑(0) ↔ (1) ↓(1)	↑(0) ↔ (2) ↓(4)	↑(0) ↔ (3) ↓(0)		↑(1) ↔ (1) ↓(2)
Sleep to SED	↑(0) ↔ (7) ↓(0)	↑(1) ↔ (1) ↓(0)	↑(4) ↔ (2) ↓(0)	↑(0) ↔ (3) ↓(0)		↑(2) ↔ (1) ↓(1)

*LPA* light physical activity, *MVPA* modertate-to-vigorous physical activity, *SED* sedentary time

↑ For the 24-h movement behaviour composition, this symbol indicates a statistically significant association. For the co-dependent associations for relative time spent in MVPA, LPA, SED, and sleep this symbol indicates a favourable association that was statistically significant. ↓ For the co-dependent associations for relative time spent in MVPA, LPA, SED, and sleep this symbol indicates an unfavourable association that was statistically significant. ↔ For the 24-h movement behaviour composition, this symbol indicates a null (non-significant) association. For the co-dependent associations for relative time spent in MVPA, LPA, SED, and sleep this symbol indicates a null (non-significant) association

**Table 3:** Summary of results of each health and developmental indicator

Component	Health and developmental indicator	
	Body composition	Bone health
Composition	↑(1) ↔ (19)	↑(0) ↔ (8)
MVPA	↑(0) ↔ (19)	↑(6) ↔ (2)
LPA	↑(2) ↔ (16) ↓(1)	↑(2) ↔ (6)
SED	↑(2) ↔ (17)	↑(1) ↔ (5) ↓(2)
Sleep	↑(1) ↔ (17) ↓(1)	↑(0) ↔ (7) ↓(1)
10% increase in component		
Others to MVPA	↑(0) ↔ (15) ↓(0)	↑(6) ↔ (2) ↓(0)
Others to LPA	↑(2) ↔ (12) ↓(1)	↑(2) ↔ (6) ↓(0)
Others to SED	↑(2) ↔ (13) ↓(0)	↑(1) ↔ (5) ↓(2)
Others to sleep	↑(2) ↔ (13) ↓(0)	↑(0) ↔ (7) ↓(1)
10% decrease in component		
MVPA to others	↑(0) ↔ (15) ↓(0)	↑(0) ↔ (2) ↓(6)
LPA to others	↑(1) ↔ (12) ↓(2)	↑(0) ↔ (2) ↓(6)
SED to others	↑(0) ↔ (13) ↓(2)	↑(2) ↔ (5) ↓(1)
Sleep to others	↑(2) ↔ (13) ↓(0)	↑(1) ↔ (7) ↓(0)

*LPA* light physical activity, *MVPA* moderate-to-vigorous physical activity, *SED* sedentary time

### Moderate-to-vigorous physical activity

Relative time spent in MVPA was favourably associated with indicators of motor skills and development (3 of 4 associations) and bone health (6 of 8 associations) (Tables 2 and 3). However, relative time in MVPA was rarely or never significantly associated with indicators of body composition (0 of 19 associations), mental health (0 of 3 associations), and social-emotional development (1 of 7 associations) (Table 2). Associations between relative time spent in MVPA with indicators of physical fitness and sleep health were not reported.

Time substitution estimates show that increasing time spent in MVPA at the expense of LPA was favourably associated with motor skills and development for half (i.e., 3 of 6) of the associations examined in the literature (Table 2). In contrast, removing time from MVPA and adding it into LPA was unfavourably associated with motor skills (3 of 6 associations) and social-emotional development (2 of 3 associations). For the other health and developmental indicators, the majority of time substitutions that involved MVPA were rated as ↔ as there were no consistent associations (Tables 4 and 5).

**Table 4:** Summary of results for the movement behavior composition and each of its components

Movement behaviour component	Health and development indicator					
	Body composition	Mental health	Motor skills and development	Social-emotional development	Fitness	Sleep
Composition	↔	↑	↑	↔	↑	↔
MVPA	↔	↔	↑	↔		
LPA	↔	↔	↔	↔		
SED	↔	↑	↔	↔		
Sleep	↔	↔	↔	↔		
Time substitutions						
MVPA to LPA	↔	↔	↓	↓	↔	↔
LPA to MVPA	↔	↔	↑	↔	↔	↔
MVPA to SED	↔	↔	↔	↓		↔
SED to MVPA	↔	↔	↔	↑	↔	↔
MVPA to sleep	↔	↔	↔	↓		↔
Sleep to MVPA	↔	↔	↔	↑	↔	↔
LPA to SED	↔	↔	↑	↔		↔
SED to LPA	↔	↔	↓	↔	↔	↔
LPA to sleep	↔	↓	↑			↔
Sleep to LPA	↔	↔	↓		↔	↔
SED to sleep	↔	↔	↓	↔		↔
Sleep to SED	↔	↔	↑	↔		↔

*LPA* light physical activity, *MVPA* moderate-to-vigorous physical activity, *SED* sedentary time

↑ Substituting time from the first movement behaviour to the second was associated with a favourable change in the health or developmental indicator. ↓ Substituting time from the first movement behaviour to the second was associated with an unfavourable change in the health or developmental indicator. ↔Substituting time from the first movement behaviour to the second was associated with a null (non-significant) change in the health or developmental indicator

**Table 5:** Summary of results for the movement behavior composition and each of its components

Movement behaviour component	Health and developmental indicator	
	Body composition	Bone health
Composition	↔	↔
MVPA	↔	↑
LPA	↔	↔
SED	↔	↔
Sleep	↔	↔
10% increase in component		
Others to MVPA	↔	↑
Others to LPA	↔	↔
Others to SED	↔	↔
Others to sleep	↔	↔
10% decrease in component		
MVPA to others	↔	↓
LPA to others	↔	↓
SED to others	↔	↔
Sleep to others	↔	↔

*LPA* light physical activity, *MVPA* moderate-to-vigorous physical activity, *SED* sedentary time

### Light physical activity

The summary presented in Table 4 indicates that relative time spent in LPA is not consistently associated with any of the health and developmental indicators examined in the literature as > 85% of the reported associations were not significant.

Results for time substitutions indicate that increasing time spent in LPA by taking it from MVPA was unfavourably associated with motor skills and development (3 of 6 associations) and indicators of socio-emotional development (2 of 3 associations). Also, increasing time spent in LPA by taking it from SED or sleep was unfavourably associated with motor skills and development (4 of 6 associations). However, taking time from LPA and reallocating it into MVPA (3 of 6 associations), SED (4 of 6 associations), or sleep (4 of 6 associations) was favourably associated with motor skills and development. Taking time from LPA and reallocating it to sleep was unfavourably associated with mental health (1 of 1 association). For the other health and developmental indicators, most time substitutions that involved LPA were rated as ↔ and there were no consistent associations.

### Sedentary time

Relative time spent in SED was mostly favourably associated with indicators of mental health (2 of 3 associations) but not indicators of body composition (2 of 19 associations), motor skills and development (0 of 4 associations), or social-emotional development (0 of 7 associations) (Tables 3 and 4). Associations between relative time spent in SED and indicators of physical fitness and sleep health were not reported.

The time substitution estimates indicate that reallocating more time into SED by taking it from LPA or sleep was favourably associated with motor skills and development (4 of 6 associations) while taking time from SED and moving it into LPA or sleep was unfavourably associated with motor skills and development (4 of 6 associations). For the other health and developmental indicators, the majority of time substitutions that involved SED were rated as ↔ (no consistent associations).

### Sleep

Relative time spent in sleep was not consistently associated with any of the health and developmental indicators examined in the literature as > 90% of the associations reported were not significant (Table 2).

Increasing time spent in sleep by taking it from LPA was favourably associated with motor skills and development (4 of 6 associations) but unfavourably associated with mental health (1 of 1 associations). Also, adding more time into sleep by removing it from SED was unfavourably associated with motor skills and development (4 of 6 associations) while taking time out of sleep and adding it into SED was favourably associated with motor skills and development (4 of 6 associations). In contrast, removing time from sleep and adding it into LPA was unfavourably associated with motor skills and development (4 of 6 associations). For the other health and developmental indicators, most time substitutions that involved sleep were rated as ↔ (no consistent associations).

## Discussion

This systematic review comprehensively examined the associations between the composition of 24-h movement behaviours and health and developmental indicators in children of the early years. The 24-h movement behaviour composition was consistently associated with indicators of mental health, motor skills and development, and physical fitness. Relative time spent in MVPA and SED were consistently associated with mental health, motor skills and development, and physical fitness. Relative time spent in LPA and sleep showed inconsistent association with the health and developmental indicators. Results for time substitutions suggest that it is favourable for some health and developmental indicators to reallocate more time into MVPA by taking it from LPA while it is unfavourable to take time out of MVPA. Taking time out of LPA and reallocating it into SED or sleep was favourable for motor skills and development while adding more time into LPA by taking it from SED or sleep was unfavourable for motor skills and development. Finally, reallocating more time into sleep by taking it from LPA was unfavourably associated with mental health. There were no consistent patterns for the other time substitutions.

We noticed a different pattern of findings in this systematic review of children of the early years than what has been observed in previous systematic reviews of school-aged children and youth and adults [13, 20]. In school-aged children and youth and adults, there is consistent evidence that the 24-h movement behaviour composition is associated with a variety of health indicators [13, 20]. Conversely, in our systematic review, the 24-h movement behaviour composition was associated with some health indicators (e.g., mental health, motor skills and development, physical fitness) but not others (e.g., body composition, bone health, social-emotional development). Furthermore, in school-aged children and youth and adults, there is consistent evidence that relative time spent in MVPA and reallocating more time into MVPA by taking time out of the other movement behaviours benefits a variety of health indicators [13, 20]. For example, two systematic reviews of adults published in 2020 concluded that the 24-h composition was associated with all of the examined health indicators and that relative time spent in MVPA was favourably associated with all examined health indicators [20]. In this review of young children, relative time spent in MVPA was only favourably associated with indicators of motor skills and development and bone health.

Several factors could explain the aforementioned age-related differences. Although body composition has been a focus of movement behaviour CoDA research in all age groups, many of the other health indicators studied in children of the early years (e.g., bone health, motor skills and development) are different from the health indicators studied in school-aged children and youth and adults (e.g., cardiometabolic risk factors). Second, the movement behaviours levels and patterns are different in children of early years than they are in older children, youth, and adults. For example, 3–5 year old Canadians accumulate 5.9 h/day of physical activity of which 19% is MVPA [30], 6–17 year old Canadians accumulate 5.3 h/day of physical activity of which 17% is MVPA [31], and Canadian adults accumulate 4.4 h/day of physical activity of which 9% is MVPA [32]. Also, unlike young children who spent a high proportion of their physical activity time in unorganized activities (i.e., unstructured free play), school-aged children youth accumulate a lot of their PA through organized and structured activities such as physical education, organized sports and programs, exercising, and active transportation [30, 33].

A few of the findings for the compositional isotemporal substitution models were counter intuitive. Specifically, increasing time spent in LPA by taking it from SED or sleep was unfavourably associated with motor skills and development (4 of 6 associations), adding more time into sleep by removing it from SED was unfavourably associated with motor skills and development (4 of 6 associations), and increasing time spent in sleep by taking it from LPA was unfavourably associated with mental health (1 of 1 associations). It is possible that these counter intuitive findings reflect some of the limitations of the studies included in this review such as spurious findings that occurred because some important confounding variables were not controlled for, the use of cross-sectional study designs that do not provide evidence of causal associations, and small sample sizes that may have increased the likelihood of type II error.

Our systematic review highlights that there are important limitations and gaps in the movement behaviour CoDA literature in young children. In addition to the small number of studies, most of the existing studies had small sample sizes with 6 of 8 having < 300 participants. This may in part explain why many of the observed associations were not significant (i.e., these studies may have been underpowered to detect modest or small effect sizes). Furthermore, only one study included infants and toddlers and more research is needed in children < 3 years old. Also, 19 of 51 (37%) of the associations examined in the literature are based on indicators of body composition, and more research is warranted for other measures of health and development. Finally, none of the studies used new CoDA strategies that aim to determine the best combinations of movement behaviours [34, 35]. In 2022, Dumuid et al. explained the concept of the “Goldilocks day” and reported that the best overall composition for health among adolescents was 10.4 h of sleep, 9.7 h of SED, 2.4 h of LIPA, and 1.5 h of MVPA [34]. Similar analyses are needed in studies of young children.

Our review is not void of limitations. It is a narrative synthesis; a meta-analysis could not be performed because there were too few studies with some parameters of interest not being reported and heterogeneity in indicator measures and time substitution approaches. Also, our systematic review was limited to papers published in the peer-reviewed literature, which might result in publication bias as studies with null findings are less likely to be published [36]. Finally, this review was limited to papers published in English. However, a recent study reported that excluding non-English publications from evidence syntheses did not change the conclusions [37].

## Conclusion

In conclusion, the 24-h movement behaviour composition was associated with indicators of mental health, motor skills and development, physical fitness but not with body composition, bone health, and social-emotional development. Future research should study 0–3 year olds, include larger sample size, consider health and development indicators other than body composition, and adopt new CoDA strategies that aim to determine the best combination of movement behaviours.

## Supplementary Information

**Additional file 1.** Supplemental materials.

## Data Availability

Group-level data were obtained from the published results of the studies included in the systematic review and are provided in the additional file.

## Abbreviations

CoDA: Compositional data analysis
PA: Physical activity
BMI: Body mass index
LPA: Light physical activity
MVPA: Moderate-to-vigorous physical activity
SED: Sedentary time
METs: Metabolic equivalents
