# Supplementary material for: A systematic review of compositional analysis studies examining the associations between sleep, sedentary behaviour, and physical activity with health indicators in early childhood
Source: J Act Sedentary Sleep Behav. 2023 Feb 1;2:1. doi: 10.1186/s44167-022-00012-2 (PMC11960365; doi:10.1186/s44167-022-00012-2)
Supplement: Supplementary file 1 — Additional file 1. Supplemental materials. [file 44167_2022_12_MOESM1_ESM.docx]

**Example of search strategy based on Ovid MEDLINE search**

Database: Ovid MEDLINE(R) ALL <1946 to December 15, 2021>

Search Strategy:

--------------------------------------------------------------------------------

1 Physical Activity.mp. (128121)

2 exp Exercise/ (223198)

3 exp Exercise Movement Techniques/ (9380)

4 exp Exercise Therapy/ (57742)

5 Physical Exertion/ (57123)

6 Motor Activity/ (99074)

7 exp Sports/ (200603)

8 (sport$ or bicycl$ or swim$ or walk$ or run$ or jog$).tw. (479159)

9 (physical$ adj2 activ$).tw. (135549)

10 (aerobic adj2 (train$ or active$)).tw. (5364)

11 "Play and Playthings"/ and (activ* or outdoor*).tw,kf. (1896)

12 ((activ* or outdoor*) adj3 play*).tw,kf. (24600)

13 playground*.tw,kf. (2033)

14 active.ti. and (space* or behavio?r* or transport* or commut* or neighbo?rhood* or park* or game* or gaming or lifestyle).mp. (17899)

15 (active adj3 (space* or behavio?r* or transport* or commut* or neighbo?rhood* or park* or game* or gaming or lifestyle)).tw,kf. (24269)

Annotation: Lines 11-15 from Sampasa-Kanyinga H, Colman I, Goldfield GS, Janssen I, Wang J, Podinic I, Tremblay MS, Saunders TJ, Sampson M, Chaput JP. Combinations of physical activity, sedentary time, and sleep duration and their associations with depressive symptoms and other mental health problems in children and adolescents: a systematic review. Int J Behav Nutr Phys Act. 2020 Jun 5;17(1):72. doi: 10.1186/s12966-020-00976-x. PMID: 32503638; PMCID: PMC7273653.

16 or/1-15 (942074)

17 Sedentary Behavior/ (11973)

18 Screen Time/ (796)

19 sedentar$.tw. (35655)

20 low energy expenditure.tw. (196)

21 (computer game* or video game* or television or tv).tw. (31929)

22 (electronic game* or gaming).tw. (4260)

23 Television/ or Computers/ or Video games/ (70536)

24 (screen based entertainment or screen-based entertainment or screen time).tw. (3033)

25 (texting or text messag* or app or apps or mobile applications).tw. (40908)

26 (smartphone* or smart phone* or cell phone* or mobile phone* or small screen*).tw. (28435)

27 (iphone* or ipad* or ipod* or tablet* or laptop* or android*).tw. (64972)

28 (social media or Facebook or Youtube or Twitter or Snapchat or Instagram or Pinterest or Skype or Vine or Weibo or reddit* or qzone).tw. (27295)

29 bed rest.mp. (7590)

30 sitting.tw. (24643)

31 (physical* adj2 inactivit*).tw. (9099)

32 (Reading/ or Books/) and ((time* or duration*).tw. or Time/ or Time Factors/) (6086)

33 or/17-32 (318992)

34 Sleep/ or sleep*.tw. (213020)

35 exp *Sleep Apnea Syndromes/ and (apnea or apnoea).ti. (22822)

36 34 not 35 (190467)

37 compositio$.ab,ti,kw. (491605)

38 16 and 33 and 36 and 37 (237)

39 "compositional data analysis".mp. (249)

40 38 or 39 (432)

41 limit 40 to "all adult (19 plus years)" (147)

42 40 not 41 (285)

43 limit 42 to (english language and yr="2015 -Current") (243)

***************************

| **Table S1:** Risk of bias assessment | | | | | | | | | |
| --- | --- | --- | --- | --- | --- | --- | --- | --- | --- |
|  | **Checklist item (Downs and Black item No.)** | **Taylor et al. (2018)** | **Carson et al. (2017)** | **Bezerra et al. (2020)** | **Kuzik et al. (2020)** | **Lemos et al. (2021)** | **McGee et al. (2019)** | **Mota et al. (2020)** | **Laurent et al. (2020)** |
| **Strength of reporting** | Is the objective of the study clearly described? (item 1) | 1 | 1 | 1 | 1 | 1 | 1 | 1 | 1 |
|  | Are the main indicators to be measured clearly described in the introduction or methods section? (item 2) | 1 | 1 | 1 | 1 | 1 | 1 | 1 | 1 |
|  | Are the characteristics of the participants included in the study clearly described? (item 3) | 1 | 1 | 1 | 1 | 1 | 1 | 1 | 1 |
|  | Are the exposure variables clearly described? (item 4 modified) | 1 | 1 | 1 | 1 | 1 | 1 | 1 | 1 |
|  | Are the covariates clearly described? (item 5 modified) | 1 | 1 | 1 | 1 | 1 | 1 | 1 | 1 |
|  | Are the main findings of the study clearly described? (item 6)^a^ | 1 | 1 | 0.5 | 1 | 0.5 | 0.5 | 0.5 | 0.5 |
|  | Does the study provide estimates of the random variability in the data for the main indicators? (item7) | 1 | 1 | 1 | 1 | 1 | 1 | 1 | 1 |
|  | Have the characteristics of participants lost to follow-up for cohort or with missing data been described? (item 9 modified)^b^ | 0 | 0 | 1 | 0 | 0) | 0 | 0 | 0 |
|  | Have 95% confidence interval and /or actual probability values been reported for the main indicators except where the probability value is <0.001? ( item 10 modified) | 1 | 1 | 1 | 1 | 1 | 1 | 1 | 1 |
| **External validity** | Were the subjects asked to participate in the study representative of the entire population from which they were recruited? (item 11) | 1 | 1 | 0 | 0 | 1 | 0 | 0 | 0 |
|  | Were those subjects who were included in the analysis representative of the entire population from which they were recruited? (item 12 modified) | 0 | 1 | 0 | 0 | 0 | 0 | 0 | 0 |
| **Internal validity (bias)** | Was an attempt made to blind those measuring the main indicators based on the exposure status? (item15 modified)^c^ | 0 | 0 | 0 | 0 | 0 | 0 | 0 | 0 |
|  | In cohort studies, do the analyses adjust for different lengths of follow-up of participants? (item 17) | 1 | N/A | N/A | N/A | N/A | N/A | N/A | N/A |
|  | Were the movement behaviours measured accurately? (added item) | 1 | 0.66 | 0.66 | 1 | 0.66 | 0.66 | 0.66 | 1 |
|  | Were the statistical tests used to assess the main indicators appropriate? (item 18)^e^ | 1 | 0.5 | 1 | 1 | 1 | 1 | 1 | 1 |
|  | Were the main indicator measures used accurate ? (item 20) | 1 | 1 | 1 | 1 | 1 | 1 | 1 | 1 |
|  | Was there adequate adjustment for confounding in the analyses from which the main findings were drawn? (item 25)^f^ | 0.5 | 0.5 | 0.5 | 1 | 0.5 | 0.5 | 0.5 | 1 |
| **Power** | Did the study have sufficient power to detect an important effect where the probability value for a difference being due to chance is <5%? (item 27) | 0 | 0 | 0 | 0 | 0 | 1 | 1 | 0 |
| ***Overall quality score*** | | *13.5/18 (good)* | *12.7/17 (good)* | *11.7/17 (fair)* | *12/17 (fair)* | *11.7/17 (fair)* | *11.7/17 (fair)* | *11.7/17 (fair)* | *11.5/17 (fair* |
| *Note*: Checklist items were scored as 1 (yes) or 0 (no) unless otherwise indicated.  ^a^ Scored as 1 when the results from both the compositional data analysis and isotemporal substitution models were both reported. Scored as 0.5 when the results from either the compositional data analysis or the isotemporal substitution models were reported. Scored as 0 when neither the results from the compositional data analysis or the isotemporal substitution models were reported.  ^b^ Scored as 1 if <10% of participants were removed because of missing data or if there were no differences between participants with complete and missing data, scored = 0 if ≥10% missing data or if there were differences between participants with complete and missing data.  ^c^ Also scored as a 0 if the checklist item question could not be answered based on the data provided in the publication.  ^d^ Scored as 0 if none of the movement behaviours were measured objectively, .33 if one movement behaviour was measured objectively, 0.66 if two movement behaviours were measured objectively, and 1 if sleep, sedentary time, and physical activity were all measured objectively.  ^e^ Scored as 0.5 if isotemporal substitution models was not conducted and presented.^f^ Scored as 0.5 if confounding was only partially adjusted for (i.e., included some but not all important confounding variables. | | | | | | | | | |

| **Table S2:**  Relationships between the movement behaviour composition and its components with health and developmental indicators | | | | | | | | | |
| --- | --- | --- | --- | --- | --- | --- | --- | --- | --- |
| **Health or developmental**  **indicator** | **Reference** | **Movement behaviour composition** | **Co-dependent association with health and developmental**  **indicator** | | | | | | |
|  |  |  | **MVPA** | | **LPA** | **SED** | | **Sleep** | |
| **Body composition** | | | | | | | | | |
| % body fat | Taylor et al. (2018), prospective at age 1 | p>.05 | 0.18 | | −1.88* | 2.94 | | − 1.24 | |
|  | Taylor et al. (2018), prospective at age 2 | p>.05 | −0.86 | | 0.53 | 0.39 | | −0.07 | |
|  | Taylor et al. (2018), prospective at age 3.5 | p>.05 | −1.35 | | 1.94 | −0.54 | | −0.05 | |
|  | Taylor et al. (2018), prospective at age 5 | p>.05 | −0.60 | | 0.41 | 0.47 | | −0.29 | |
| Fat free mass | Taylor et al. (2018), prospective at age 1 | p>.05 | 0.03 | | 0.16 | 0.27 | | − 0.46 | |
|  | Taylor et al. (2018), prospective at age 2 | p>.05 | 0.09 | | −0.37 | 0.6 | | −0.32 | |
|  | Taylor et al. (2018), prospective at age 3.5 | p>.05 | 0.06 | | 1.26* | 0.87 | | −2.19* | |
|  | Taylor et al. (2018), prospective at age5 | p>.05 | 0.17 | | 0.48 | 0.17 | | −0.82 | |
| Waist circumference | Carson et al. (2017) | p=.72 | 0.08 | | 0.23 | 0.87 | | −1.95 | |
| BMI | Carson et al. (2017) | p=.006* | −0.09 | | 0.62 | 0.19 | | −0.72 | |
|  | Taylor et al. (2018), cross-sectional at age 1 | p>.05 | −0.06 | | 0.03 | 0.02 | | 0 | |
|  | Taylor et al. (2018), cross-sectional at age 2 | p>.05 | −0.09 | | −0.08 | −0.28 | | 0.45 | |
|  | Taylor et al. (2018), cross-sectional at age 3.5 | p>.05 | −0.17 | | 0.77* | 0.97* | | −1.57* | |
|  | Taylor et al. (2018), cross-sectional at age 5 | p>.05 | 0.09 | | 0.55 | 0.25 | | −0.89 | |
|  | Taylor et al. (2018), prospective at age 1 | p>.05 | 0.03 | | −0.08 | 0.28 | | −0.24 | |
|  | Taylor et al. (2018), prospective at age 2 | p>.05 | 0.03 | | −0.05 | 0.45 | | −0.43 | |
|  | Taylor et al. (2018), prospective at age 3.5 | p>.05 | 0.09 | | 0.23 | −0.24 | | −0.08 | |
|  | Kuzik et al. (2020) | p=.22 | 0.65 | | -1.07 | -0.65 | | 1.07 | |
|  | McGee et al. (2019) | NR | 0.6 | | 0.1 | 1.0 | | -1.0 | |
| **Bone health** |  |  |  | |  |  | |  | |
| Bone mineral density | Taylor et al. (2018), prospective at age 1 | p>.05 | −0.001 | | 0.024* | − 0.032* | | 0.01 | |
|  | Taylor et al. (2018), prospective at age 2 | p>.05 | 0.012* | | 0.002 | −0.010 | | −0.003 | |
|  | Taylor et al. (2018), prospective at age 3.5 | p>.05 | 0.014* | | 0.015 | −0.007 | | −0.021 | |
|  | Taylor et al. (2018), prospective at age 5 | p>.05 | 0.019* | | −0.001 | 0.036 | | −0.054* | |
| Bone mineral content | Taylor et al. (2018), prospective at age 1 | p>.05 | −3.2 | | 42.6* | − 88.7* | | 49.3 | |
|  | Taylor et al. (2018), prospective at age 2 | p>.05 | 23.9* | | 3.1 | −46.6 | | 19.6 | |
|  | Taylor et al. (2018), prospective at age 3.5 | p>.05 | 26.0* | | 16.9 | −26.0 | | −16.9 | |
|  | Taylor et al. (2018), prospective at age 5 | p>.05 | 38.0* | | −19.6 | 75.1* | | −93.5 | |
| **Mental health** |  |  |  | | | | | | |
| Executive function | Bezerra et al. (2020) | p<.0001* | NR | NR | | | NR | | NR |
| Response inhibition | Kuzik et al. (2020) | p=.07 | 0.08 | | -0.1 | 0.27 * | | -0.26 | |
| Working memory | Kuzik et al. (2020) | p=.01* | -0.33 | | 0.88 | 0.78 | | -1.33 | |
| Vocabulary | Kuzik et al. (2020) | p=<.01* | 2.96 | | -4.44 | 10.04 * | | -8.56 | |
| **Motor skills and development** |  |  |  | |  |  | |  | |
| Locomotor skills | Kuzik et al. (2020) | p=.02* | 9.0* | | -14.5 | 9.3 | | -3.8 | |
| Object motor skills | Kuzik et al. (2020) | p=.001* | 12.4* | | -14.2* | -0.5 | | 2.3 | |
| Total motor skills | Kuzik et al. (2020) | p=.001* | 21.4 * | | -28.8* | 8.7 | | -1.4 | |
| Locomotor skills | Mota et al. (2020) | p<.001* | NR | | NR | NR | | NR | |
| Object motor skills | Mota et al. (2020) | p<.001 | NR | | NR | NR | | NR | |
| Total motor skills | Mota et al. (2020) | p<.001* | NR | | NR | NR | | NR | |
| **Social-emotional development** |  |  |  | |  |  | |  | |
| Behavioural self-regulation | Kuzik et al. (2020) | p=.98 | -0.07 | | -0.1 | -0.07 | | 0.24 | |
| Cognitive self-regulation | Kuzik et al. (2020) | p=.15 | 0.52 | | -1.18 | 0.17 | | 0.48 | |
| Emotional self-regulation | Kuzik et al. (2020) | p=.90 | -0.14 | | 0.89 | -0.21 | | -0.53 | |
| Externalizing symptoms | Kuzik et al. (2020) | p=.74 | 0.37 | | -0.71 | 0.34 | | -0.00 | |
| Internalizing symptoms | Kuzik et al. (2020) | p=.32 | -0.2 | | -0.04 | 0.11 | | 0.13 | |
| Sociability | Kuzik et al. (2020) | p=.05 | 0.71 * | | -0.64 | -0.00 | | -0.08 | |
| Prosocial behaviour | Kuzik et al. (2020) | p=.97 | 0.31 | | -0.5 | 0.42 | | -0.22 | |
| **Physical fitness** |  |  |  | |  |  | |  | |
| Cardiorespiratory fitness | Lemos et al. (2021) | p=.007* | NR | | NR | NR | | NR | |
| Speed-agility | Lemos et al. (2021) | p<.001* | NR | | NR | NR | | NR | |
| Lower-body strength | Lemos et al. (2021) | p=.01* | NR | | NR | NR | | NR | |
| **Sleep health** |  |  |  | |  |  | |  | |
| Sleep efficiency | Laurent et al. (2020) | p<.001* | NR | | NR | NR | | NR | |
| Nap frequency | Laurent et al. (2020) | p=.003* | NR | | NR | NR | | NR | |
| Total sleep disturbances score | Laurent et al. (2020) | p=.1 | NR | | NR | NR | | NR | |
| Bedtime resistance score | Laurent et al. (2020) | p=.07 | NR | | NR | NR | | NR | |
| LPA, light physical activity; MVPA, moderate-to-vigorous physical activity; NR, not reported; SED, sedentary time.  * statistically significant (p<.05) | | | | | | | | | |

| **Table S3:** Relationships between the movement behaviour time reallocations and health and developmental indicators. | | | | | | | | | | | | | | |  |  |  |  |  |  |
| --- | --- | --- | --- | --- | --- | --- | --- | --- | --- | --- | --- | --- | --- | --- | --- | --- | --- | --- | --- | --- |
| **Health and developmental indicator** | **Reference** | **Time reallocation** | **Time reallocated from the first listed movement behaviour to the second listed movement behaviour** | | | | | | | | | | | |  |  |  |  |  |  |
|  |  |  | **MVPA to LPA** | **LPA to MVPA** | **MVPA to SED** | **SED to MVPA** | **MVPA to sleep** | **Sleep to MVPA** | **LPA to SED** | **SED to LPA** | **LPA to sleep** | **Sleep to LPA** | **SED to sleep** | **Sleep to SED** |  |  |  |  |  |  |
| **Body composition** | | | | | | | | | | | | | | |  |  |  |  |  |  |
| BMI | McGee et al. (2019) | 30 min | 0.24 | −0.17 | 0.13 | −0.05 | 0.21 | −0.14 | −0.12 | 0.11 | −0.04 | 0.03 | 0.08 | −0.08 |  |  |  |  |  |  |
|  | Kuzik et al. (2020) | 20 min | NS | NS | -0.23 | 0.19 | NS | NS | NS | NS | NR | NR | NS | NS |  |  |  |  |  |  |
| Triceps skinfold | McGee et al. (2019) | 30 min | 0.01 | 0.01 | 0.01 | 0 | 0.05 | −0.04 | 0.01 | 0 | 0.05 | −0.05 | 0.04 | −0.04 |  |  |  |  |  |  |
| Subscapular skinfold | McGee et al. (2019) | 30 min | 0.11 | −0.08 | 0.08 | −0.05 | 0.07 | −0.04 | −0.03 | 0.03 | −0.04 | 0.03 | 0 | 0 |  |  |  |  |  |  |
| % body mass | McGee et al. (2019) | 30 min | −0.02 | 0.14 | 0.28 | −0.20 | 0.29 | −0.20 | 0.33 | −0.30 | 0.33 | −0.31 | 0 | −0.01 |  |  |  |  |  |  |
| Fat mass | McGee et al. (2019) | 30 min | 0.03 | −0.02 | 0.06 | −0.04 | 0.04 | −0.02 | 0.02 | −0.02 | 0.01 | −0.01 | −0.02 | 0.02 |  |  |  |  |  |  |
| Fat-free mass | McGee et al. (2019) | 30 min | 0.11 | −0.13 | −0.08 | 0.08 | 0.01 | −0.01 | −0.21* | 0.2* | −0.12 | 0.11 | 0.09 | −0.09 |  |  |  |  |  |  |
|  |  |  |  |  |  |  |  |  |  |  |  |  |  |  |  |  |  |  |  |  |
| **Mental health** | | | | | | | | | | | | | | |  |  |  |  |  |  |
| Executive function | Bezerra et al. (2020) | 5 min | 0.35* | −0.21 | 0.14 | −0.21 | 0.06* | −0.17* | −0.19 | 0.02 | −0.36* | 0.19 | −0.15 | −0.02 |  |  |  |  |  |  |
| Vocabulary | Kuzik et al. (2020) | 30 min | NS | NS | NS | NS | NS | NS | NS | -1.11 | NR | NR | -1.08 | 1.03 |  |  |  |  |  |  |
| **Motor skills and development** | | | | | | | | | | | | | | |  |  |  |  |  |  |
| Locomotor skills | Mota et al. (2020) | 20 min | -2.04* | 1.81* | -0.92 | 0.59 | -1.26 | 0.95 | 1.2* | -1.14* | 0.86* | -0.78* | -0.33* | 0.35* |  |  |  |  |  |  |
| Object motor skills | Mota et al. (2020) | 20 min | -0.74 | 0.65 | -0.35 | 0.23 | -0.47 | 0.36 | 0.42 | -0.4 | 0.3 | -0.27 | -0.13 | 0.13 |  |  |  |  |  |  |
| Total motor skills | Mota et al. (2020) | 20 min | -1.3* | 1.15* | -0.57* | 0.36 | -0.79 | 0.59 | 0.78* | -0.74* | 0.56* | -0.51* | -0.23* | 0.22* |  |  |  |  |  |  |
| **Social-emotional development** | | | | | | | | | | | | | | |  |  |  |  |  |  |
| Cognitive self-regulation | Kuzik et al. (2020) | 30 min | -0.25 | 0.22 | NS | NS | NS | NS | NS | NS | NR | NR | NS | NS |  |  |  |  |  |  |
| Internalizing | Kuzik et al. (2020) | 30 min | NS | NS | † | ‡ | † | ‡ | NS | NS | NR | NR | NS | NS |  |  |  |  |  |  |
| Sociability | Kuzik et al. (2020) | 30 min | -0.26 | † | -0.21 | 0.16 | -0.21 | 0.16 | NS | NS | NR | NR | NS | NS |  |  |  |  |  |  |
| **Fitness** | | | | | | | | | | | | | | |  |  |  |  |  |  |
| Cardiorespiratory fitness | Lemos et al. (2021) | 15 min | -2.04 | 1.61 | NR | 1.59* | NR | 1.83* | NR | -0.03 | NR | 0.21 | NR | NR |  |  |  |  |  |  |
| Speed-agility | Lemos et al. (2021) | 15 min | -2.63 | 2.39 | NR | 1.32 | NR | 0.8 | NR | -0.99 | NR | -1.52* | NR | NR |  |  |  |  |  |  |
| Muscular strength | Lemos et al. (2021) | 15 min | -0.35 | 0.09 | NR | 0.43 | NR | 1.44 | NR | 1.31 | NR | 1.31* | NR | NR |  |  |  |  |  |  |
| **Sleep health** | | | | | | | | | | | | | | |  |  |  |  |  |  |
| Sleep efficiency | Laurent et al. (2020) | 30 min | 0.43 | −0.45 | 0.42* | −0.45* | −0.31 | 0.34 | −0.04 | −0.03 | −0.78* | 0.76* | −0.77* | 0.75* |  |  |  |  |  |  |
| Nap frequency | Laurent et al. (2020) | 30 min | −0.13 | 0.16 | −0.02 | 0.04 | 0.2 | −0.19 | 0.12 | −0.10 | 0.35* | −0.33* | 0.23* | −0.23* |  |  |  |  |  |  |
| Total sleep disturbances | Laurent et al. (2020) | 30 min | −0.95 | 0.68 | −1.16* | 0.92* | −1.22 | 0.98 | −0.22 | 0.22 | −0.29 | 0.28 | −0.05 | 0.07 |  |  |  |  |  |  |
| Bedtime resistance | Laurent et al. (2020) | 30 min | 0.03 | −0.06 | −0.03 | 0.01 | −0.16 | 0.15 | −0.08 | 0.06 | −0.21 * | 0.2* | −0.13* | 0.13* |  |  |  |  |  |  |

LPA, light physical activity; NR = not reported; NS = non-significant (p>.05); MVPA, moderate-to-vigorous physical activity; SED, sedentary time

* significant association (p<.05)

†significant (p<.05) positive association after removing influential participants

‡ significant (p<.05) negative association after removing influential participants

| **Table S4:** Relationships between the movement behaviour time reallocations and health and developmental indicators. | | | | | | | | | | |
| --- | --- | --- | --- | --- | --- | --- | --- | --- | --- | --- |
| **Health and devleop-mental indicator** | **Reference** |  | **10% increase in component** | | | | **10% decrease in component** | | | |
|  |  |  | **Others to MVPA** | **Others to LPA** | **Others to SED** | **Others to sleep** | **MVPA to others** | **LPA to others** | **SED to others** | **Sleep to others** |
| **Body composition** | | | | | | | | | | |
| BMI | Taylor et al. (2018), cross-sectional at age 1 | | −0.004 | 0.01 | −0.008 | −0.001 | 0.004 | −0.011 | 0.008 | 0.001 |
|  | Taylor et al. (2018), cross-sectional at age 2 | | −0.007 | −0.009 | −0.035 | 0.074 | 0.008 | 0.009 | 0.037 | −0.075 |
|  | Taylor et al. (2018), cross-sectional at age 3.5 | | −0.014 | 0.081* | 0.122* | −0.248* | 0.016 | −0.087* | −0.129* | 0.252* |
|  | Taylor et al. (2018), cross-sectional at age 5 | | 0.008 | 0.057 | 0.032 | −0.137 | −0.008 | −0.061 | −0.034 | 0.14 |
|  | Taylor et al. (2018), prospective at age 1 | | 0.003 | 0.002 | 0.024 | −0.038 | −0.003 | −0.002 | −0.024 | 0.038 |
|  | Taylor et al. (2018), prospective at age 2 | | 0.003 | −0.006 | 0.056 | −0.071 | − 0.003 | 0.006 | −0.059 | 0.072 |
|  | Taylor et al. (2018), prospective at age 3.5 | | 0.008 | 0.024 | −0.031 | −0.013 | −0.009 | −0.026 | 0.032 | 0.013 |
| Fat-free mass index at 5 years | Taylor et al. (2018), prospective at age 1 | | 0.003 | 0.015 | 0.038 | −0.079 | −0.003 | −0.017 | −0.039 | 0.079 |
|  | Taylor et al. (2018), prospective at age 2 | | 0.008 | −0.039 | 0.075 | −0.053 | −0.008 | 0.042 | −0.079 | 0.053 |
|  | Taylor et al. (2018), prospective at age 3.5 | | 0.005 | 0.132* | 0.11* | −0.346* | − 0.005 | −0.143* | −0.116* | 0.352* |
|  | Taylor et al. (2018), prospective at age 5 | | 0.015 | 0.05 | 0.022 | −0.127 | −0.016 | −0.054 | −0.023 | 0.129 |
| % body fat at 5 years | Taylor et al. (2018), prospective at age 1 | | 0.01 | −0.18* | 0.41 | −0.21 | −0.02 | 0.19* | −0.42 | 0.21 |
|  | Taylor et al. (2018), prospective at age 2 | | −0.07 | 0.06 | 0.05 | −0.01 | 0.08 | −0.06 | −0.05 | 0.01 |
|  | Taylor et al. (2018), prospective at age 3.5 | | −0.11 | 0.2 | −0.07 | −0.01 | 0.13 | −0.22 | 0.07 | 0.01 |
|  | Taylor et al. (2018), prospective at age 5 | | −0.05 | 0.04 | 0.06 | −0.04 | 0.06 | −0.05 | −0.06 | 0.04 |
| **Bone health** | | | | | | | | | | |
| Bone mineral density | Taylor et al. (2018), prospective at age 1 | | 0 | 0.002* | −0.004* | 0.002 | 0 | −0.002* | 0.005* | −0.002 |
|  | Taylor et al. (2018), prospective at age 2 | | 0.001* | 0 | −0.001 | −0.001 | −0.001* | 0 | 0.001 | 0.001 |
|  | Taylor et al. (2018), prospective at age 3.5 | | 0.001* | 0.002 | −0.001 | −0.003 | −0.001* | −0.002 | 0.001 | 0.003 |
|  | Taylor et al. (2018), prospective at age 5 | | 0.002* | 0 | 0.005 | −0.008* | −0.002* | 0 | −0.005 | 0.009* |
| Bone mineral content at 5 years | Taylor et al. (2018), prospective at age 1 | | −0.3 | 4.0* | − 12.3* | 8.4 | 0.3 | −4.4* | 12.8* | − 8.4 |
|  | Taylor et al. (2018), prospective at age 2 | | 2* | 0.3 | −5.8 | 3.2 | −2.2* | −0.4 | 6.1 | −3.3 |
|  | Taylor et al. (2018), prospective at age3.5 | | 2.2* | 1.8 | −3.3 | −2.7 | −2.4* | −1.9 | 3.5 | 2.7 |
|  | Taylor et al. (2018), prospective at age 5 | | 3.2* | −2.0 | 9.6* | −14.4 | −3.5* | 2.2 | −10.1* | 14.7 |

LPA, light physical activity; MVPA, moderate-to-vigorous physical activity; SED, sedentary time

* statistically significant (p<.05)
